# A simple mechanical technique to obtain carbon nanoscrolls from graphite nanoplatelets

**DOI:** 10.1186/1556-276X-8-403

**Published:** 2013-09-30

**Authors:** Gianfranco Carotenuto, Angela Longo, Sergio De Nicola, Carlo Camerlingo, Luigi Nicolais

**Affiliations:** 1 Institute for Composite and Biomedical Materials, National Research Council, Viale Kennedy, 54 - Mostra d'Oltremare Pad. 20, Naples 80125, Italy; 2 SPIN Institute, National Research Council, Complesso Universitario di M.S. Angelo, Via Cinthia, Naples 80126, Italy; 3 INFN Sezione di Napoli, Complesso Universitario di M.S. Angelo, Via Cinthia, Naples 80126, Italy; 4 Institute of Cybernetics “E. Caianiello”, National Research Council, Via Campi Flegrei 34, Pozzuoli 80078, Italy; 5 Department of Material Engineering and Production, University of Naples “Federico II”, Piazzale Tecchio, 80, Naples 80125, Italy

**Keywords:** Carbon nanoscrolls, Shear stress, Bi-axially oriented polypropylene, Graphite nanoplatelets

## Abstract

A simple approach for the bulk production of carbon nanoscrolls (CNSs) is described. This method is based on the application of shear-friction forces to convert graphite nanoplatelets into carbon nanoscrolls using a bi-axially oriented polypropylene (BOPP) surface. The combined action of shear and friction forces causes the exfoliation of graphite nanoplatelets and the simultaneous roll-up of graphite layers. Evidence of the CNS formation is given by optical microscopy, scanning electron microscopy, and transmission electron microscopy. These investigations reveal that the CNSs have a long tube-like and fusiform structure with a hollow core surrounded by few layers of graphene. Micro-Raman spectroscopy shows that the produced structures are not defect free, and optical spectroscopy reveals distinctive features due to the presence of two weak absorption bands at 224 and 324 nm.

## Background

Graphene molecules were first extracted from a graphite crystal by a simple micromechanical approach (mechanical cleavage) [1,2]. During the graphite crystal peeling out process, the applied mechanical stress causes the separation of the graphene layers, contrasting the interlayer interaction forces. This procedure is known as the Scotch type or drawing method since the mechanical exfoliation resembles writing with a pencil. This method has allowed obtaining graphene in sufficient quantities for investigating fundamental physics. Since the initial discovery, different experimental approaches and chemical synthesis methods have been applied to obtain graphene sheets to be subsequently used to fabricate various devices and materials for specific technological applications. Considerable attention has been paid to the observed significant deviation undergone by the graphene sheets from planar geometry [3]. The formation of ripples with local curvature, membranes, ribbons, and scrolled structures raises many problems, both from the theoretical and the experimental point of view, such as what are the governing parameters and what role they play in determining the conformational changes in a low-dimensional material such as graphene, and to which extent it is possible to control the occurrence of these morphological variations to achieve the goal of producing and assembling high-quality structures for large-scale graphene applications. Scrolled graphene sheets are very important carbon nanostructures that offer a number of useful physical characteristics (e.g., very high specific surface area, and electrical and thermal conductivity), adequate for applications in different technological fields like, for example, sorbents, catalyst supports, highly porous electrodes for batteries and supercapacitors, hydrogen storage materials, fillers for high-strength structural composites, etc. [4,5].

## Methods

In this letter we report on a simple and very effective way of fabricating carbon nanoscrolls (CNSs) [6-10] from graphite nanoplatelets (GNPs). This preparation method is based on a shear-friction mechanism to transform GNPs to high-quality CNSs with high yield. A shear stress acting on the graphite nanoplatelets causes a relative slip of the carbon layers which move over each other, resulting in a complete exfoliation of the graphite nanocrystal. The coupling between adjacent graphene layers in the nanocrystalline graphite crystals gets weaker as the thickness of these nanoplatelets decreases. Therefore, since the graphene sheets at the surface of the graphite nanocrystal are weakly bonded together, their sliding and separation take place easily under the action of weak shear forces [11]. However, the shear-friction mechanism for fabricating CNSs is twofold. When the shear-induced mechanical exfoliation takes place and the graphene sheets slide against a rough surface, a rolling-up process occurs under the combined action of shear and friction forces, leading to the formation of nanoscroll structures. The presence of a nanofibrous surface plays a crucial role. A rolling-up process with noticeable formation of CNSs has been observed under shear-friction on a bi-axially oriented polypropylene (BOPP) substrate. The shear-induced exfoliation process without the concurrent action of the friction force did not result in the formation of CNSs. It was found that under exfoliation of GNP against a low-density polyethylene (LDPE) surface, graphene sheets distributed and covered quite uniformly the surface of the film. On the other end, the GNP exfoliation against a BOPP surface resulted in massive formation of scrolled structures. This different behavior is ascribed to the presence of friction which is more effective in the latter case. In fact, the roughness of BOPP is 4.20 Å [12,13], comparable to the graphite interlayer spacing (3.354 Å), thus leading to enhanced mechanical grip between the two sliding surfaces.

## Results and discussion

**Figure 1:**
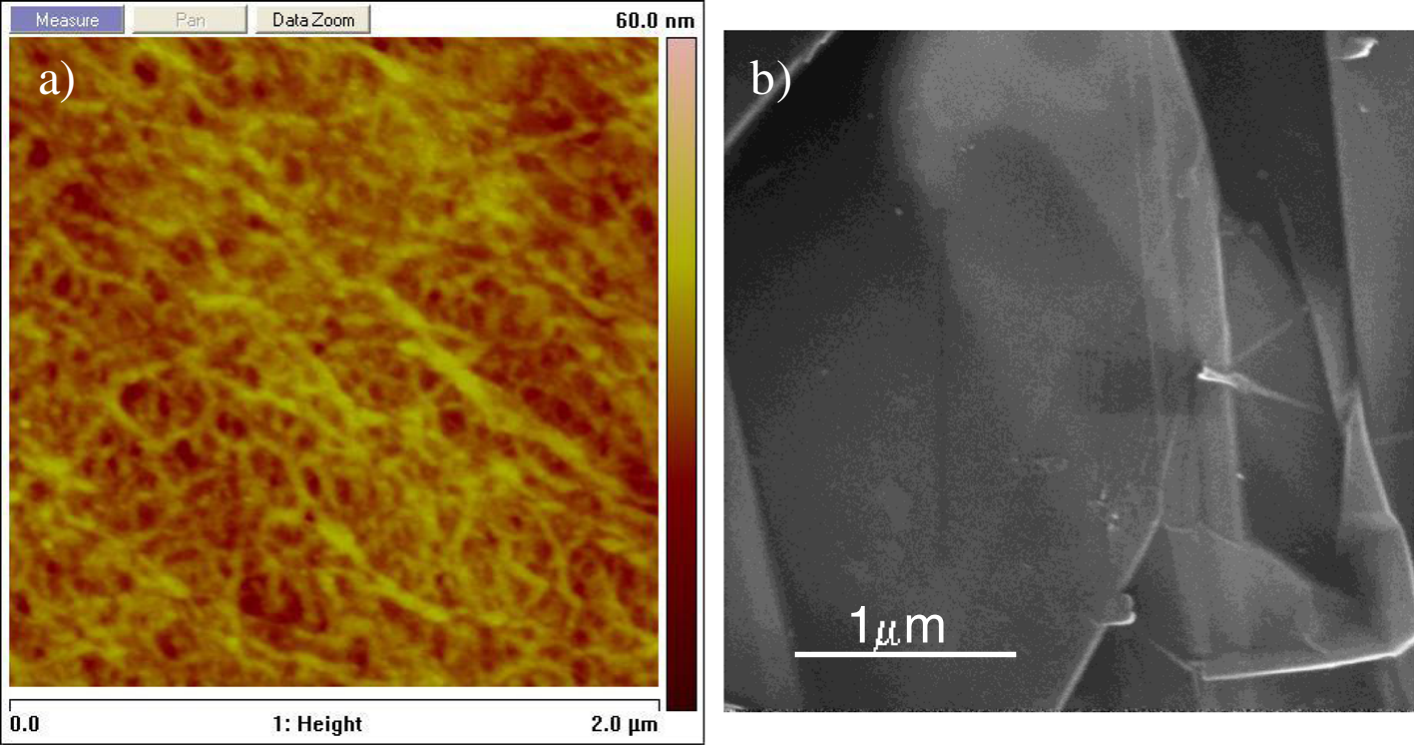
AFM image of the BOPP film nanoporous surface (a) and SEM micrograph of the GNP precursor (b).

The role of shear-stress forces in the treatment of graphite by ball-milling technology has been previously suggested as an explanation for the occasional formation of nanoscrolls [14]. However, a very limited amount of low-quality CNS results at the end of the graphite grinding process. On the other hand, to the best of our knowledge, we are the first to achieve a massive production of well-formed CNSs by applying a combination of shear stress and friction forces to a GNP sample in a very simple technique that does not require the use of any special apparatus. In particular, an alcoholic (ethanol, 99.9%, Aldrich, St. Louis, MO, USA) dispersion of GNP was prepared, according to our previously developed experimental procedure [15,16]. This dispersion was slowly rubbed on the surface of a BOPP film (with a thickness of 40 μm, Manucor S.p.A., Sessa Aurunca, Italy) using a LDPE piece. The suspension was allowed to dry during the rubbing process. After drying, the concentrated liquid suspension was removed from the BOPP film by pouring ethanol on it. The resulting black suspension contained a large amount of nanoscrolls. Nanoscrolls can be separated from the unrolled and/or partially rolled graphene-based material by sedimentation in ethanol since their Stokes coefficient value is significantly higher than that for graphene sheets. The nanofibrous structure of the BOPP film surface can be conveniently imaged by atomic force microscopy (AFM; see Figure 1a) [17]. As visible, the BOPP surface is made of nanosized polypropylene fibers that provide the resistant friction force inducing the separation of the graphite nanocrystal edges, thus causing a rolling-up process under the concomitant action of the applied shear stress.

**Figure 2:**
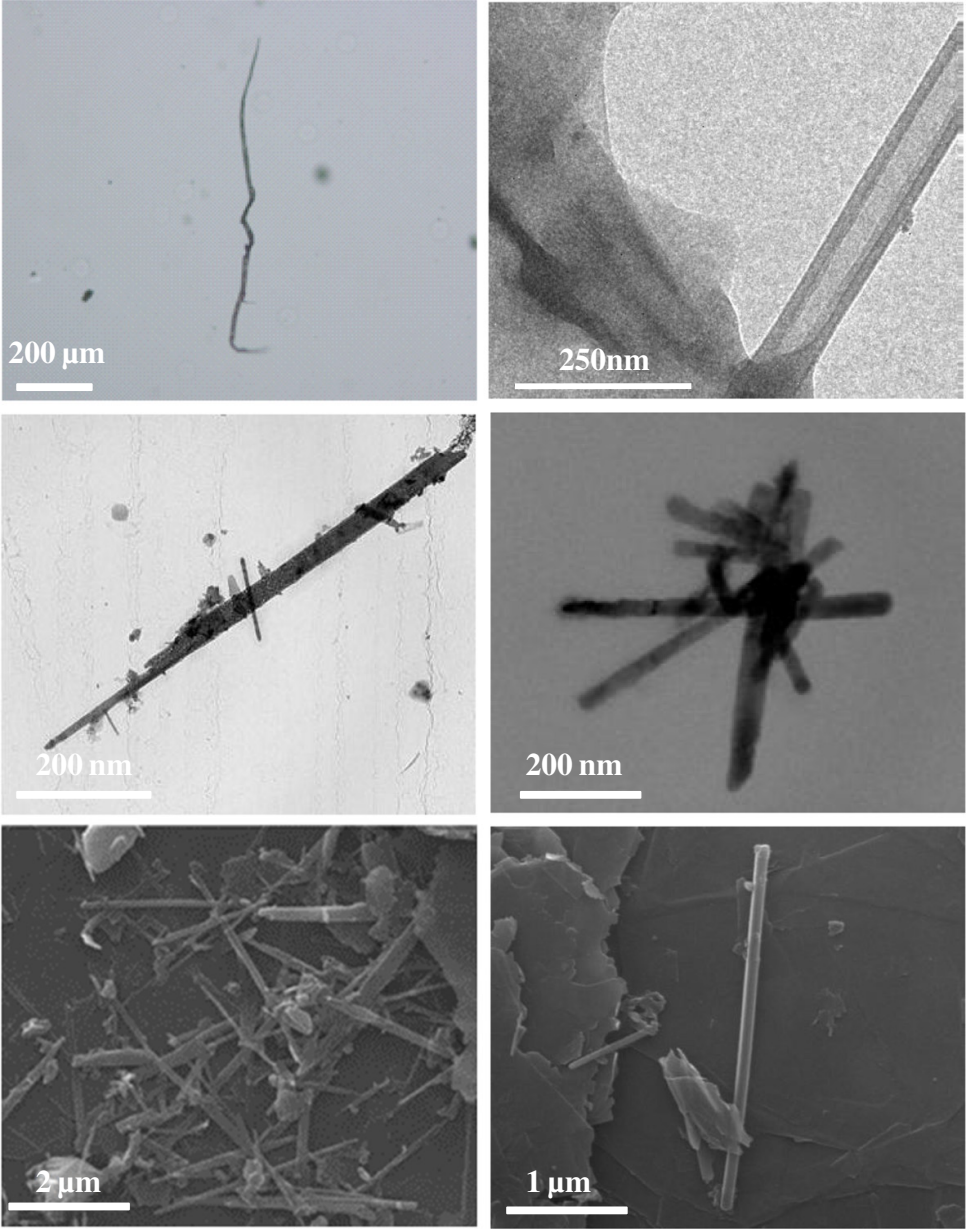
OM, TEM, and SEM micrographs of the produced carbon nanoscrolls (from top to bottom).

The typical morphology of a GNP sample used as a precursor in the CNS fabrication process is shown in Figure 1b. As visible, the starting carbon material contained only flat graphite nanoplatelets with sharp edges. The GNP unities have two main dimensions of a few microns and are characterized by an average thickness of 20 nm. After the simultaneous application of shear and friction forces, the material morphology resulted to be significantly modified. As visible in Figure 2, a large amount of tubular structures was generated by the rolling-up of the graphene monolayers generated during the GNP exfoliation. In particular, these carbon nanoscrolls are structurally made by continuous graphene sheets rolled-up in a tube-like structure with a hollow core, resembling a multi-walled carbon nanotube [18]. However, a number of morphologies are produced by this mechanical approach; in fact, the graphene monolayers, generated from the GNP exfoliation, can roll in different ways under the effect of the applied shear-friction force. Cylindrical and fusiform nanoscroll structures are usually found together with partially rolled, multi-rolled, and other irregularly shaped rolled structures. In addition, carbon nanoscrolls characterized by a significant length (few hundred microns) are not stereo-rigid and appear like a sort of hair since they are bended in different points by the presence of defects (narrowing) along their structure.

Cylindrical nanoscrolls have very uniform diameters and tend to form bundles like carbon nanotubes because of π-π interactions (see the transmission electron microscopy (TEM) micrograph given in Figure 2). Typical lengths, *L*, of the produced cylindrical nanoscrolls range from 0.5 to 2.5 μm, and the diameter, *D*, is *ca.* 100 nm. Consequently, each cylindrical nanoscroll should contain from two to eight inner layers, *N = L / πD*. In Additional file 1, a more precise calculation of the inner layer number is reported, considering an Archimedean spiral-type structure.

Nanoscrolls containing only a few graphene layers result to be quite transparent (see the scanning electron microscopy (SEM) micrographs in Figure 2). However, for fusiform nanoscrolls, the number of layers is greater by a factor √2 compared to that for cylindrical nanoscrolls. For a length *L* = 2.5 μm, we have *N = L√*2 */ πD* (approximately 11). Both cylindrical and fusiform carbon nanoscrolls are hollow, and therefore, they might be of particular interest for many technological applications like hydrogen storage, drug delivery, novel composite nanomaterial fabrication, etc.

**Figure 3:**
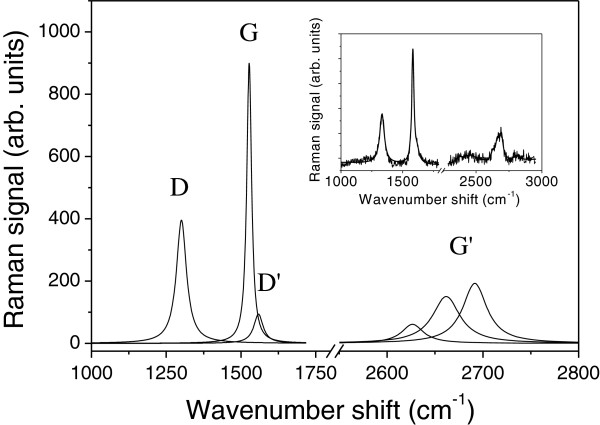
**Deconvolution of the CNS Raman spectrum at 632.8 nm in Lorentzian components.** The Raman and curve fitting signals are shown in the inset.

The produced CNSs have been characterized by micro-Raman spectroscopy (Horiba Jobin-Yvon TriAx monochromator (Kyoto, Japan), equipped with a liquid-nitrogen-cooled charge-coupled detector and a grating of 1,800 grooves/mm, which allows a final spectral resolution of 4 cm^−1^). Raman spectroscopy has been widely used as a fast, powerful, and nondestructive method for characterizing *sp*^2^ carbon systems and can provide information about the defects of the structure. Results of the micro-Raman spectroscopy scattering measurements carried out on the CNSs fabricated by the shear-friction method are shown in Figure 3. The spectra were recorded under ambient condition using a He-Ne (632.8 nm) laser source. The laser light was focused to a 1- to 2-μm spot size on the samples under low-power irradiation to avoid additional heating effect during the measurement. The deconvolution of the Raman signal in Lorentzian components points out the occurrence of sharp features at 1,583 cm^−1^ (G-band), 1,332 cm^−1^ (D-band), and 1,617 cm^−1^ (D'-band). These Raman modes are typical of disordered graphene [19] and of carbon nanoscrolls [18,20,21]. The D and D' modes are dispersive bands, and hence, their actual position and intensity depend on the laser excitation energy [19]. Their intensity with respect to that of the G mode is relevant but comparable to the data reported in [20] for Raman spectroscopy at *λ* = 632.8 nm. This feature indicates the presence of a disorder in the graphene layer, presumably more significant at the edges where the translational symmetry is broken. Since nanoscrolls have considerable edge length, a significant contribution of D and D' modes to the Raman signal is expected [20]. The broad G' mode, due to second-order Raman scattering, is centered at about 2,687 cm^−1^, blue-shifted with respect to the value expected for monolayer graphene (*ca.* 2,660 cm^−1^). As it is shown in Figure 3, the G' mode peak can be accounted by three Lorentzian peaks centered at 2,627, 2,662, and 2,692 cm^−1^, respectively. Because the intensity of the mode at 2,692 cm^−1^ increases with the increase of the graphene layer number [22], the estimated number of coils in the investigated CNS is 5, which is in good agreement with the numerical value derived from morphological considerations.

**Figure 4:**
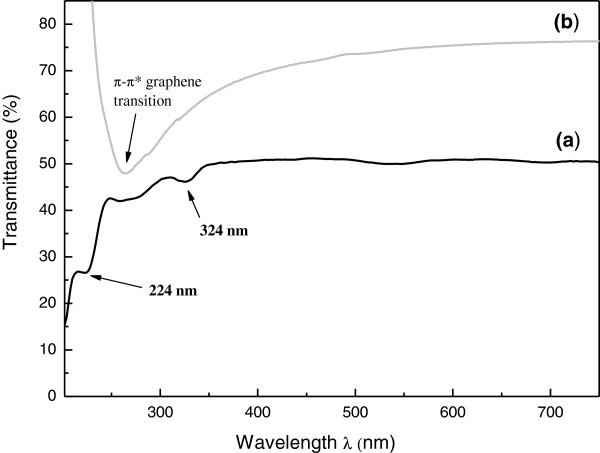
UV–vis spectrum of carbon nanoscrolls (a) and graphene (b).

The characterization of CNSs by optical absorption spectroscopy (UV–vis) shows some interesting features which are a clear evidence of the conformational modifications of the graphene sheets. A comparison between the typical UV–vis absorption spectrum of the fabricated CNSs and that of a thin graphene layer (supported on a LDPE film) is shown in Figure 4. The graphene UV–vis spectrum exhibits a single pronounced absorption band in the UV region at 264 nm and a flat absorption band over the visible region resulting from the linear dispersion of Dirac electrons in graphene. The band at 264 nm is produced by the collective π → π* electronic transition of the condensed aromatic rings in the graphene sheet [23].

This band is red-shifted at a wavelength of 324 nm in the absorption spectrum of carbon nanoscrolls, and it is quite broad and of low intensity. This red shift is probably caused by the in-phase mode for the electric field polarization of adjacent graphene sheets present in the rolled structure of the nanoscrolls. The broad signal of low intensity at 263 to 275 nm is probably due to the residual unrolled graphene sheets present in the sample. Furthermore, there is an additional ultraviolet absorption band at 224 nm which may be ascribed to possible excitation of transverse modes.

## Conclusions

In conclusion, we have developed a very simple technique to produce bulk quantities of CNSs. The method makes use of a shear-friction mechanism to transform graphite nanoplatelets to carbon nanoscrolls, employing a nanofibrous bi-axially oriented polypropylene surface. The combined action of shear and friction forces causes exfoliation of graphite nanoplatelets and the simultaneous roll-up of graphite layers. TEM studies show that the fabricated CNSs have a long tubular and fusiform structure with a hollow core surrounded by few layers of graphene. The Raman spectrum of the CNSs indicates that the structures are incompletely defect free. Optical spectroscopy shows the presence of additional absorption bands compared to the spectrum of graphene. These carbon nanomaterials are very useful structures that offer a number of advantages compared to planar graphene, and this work is very helpful for exploring a new synthesis methodology for CNS massive production.

## Competing interests

The authors declare that they have no competing interests.

## Authors’ contributions

GC conceived of the experimental design and co-wrote the paper. AL participated in the design of the experiment and developed the sample preparation. SDN developed the theoretical model and co-wrote the paper. CC performed the Raman measurements and co-wrote the paper. LN participated in the design of the experiment and coordination. All authors read and approved the final manuscript.

## Authors’ information

GC is a senior researcher at the Institute for Composite and Biomedical Materials, Italian National Research Council. His present research interests are in the field of advanced functional materials based on polymer-embedded inorganic nanostructures. In particular, his activity concerns the development of new chemical routes for the controlled synthesis of metal and semiconductor clusters in polymeric matrices, the fabrication of devices based on properties of nanoscopic objects (luminescence of quantum dots, tunable surface plasmon absorption of nanosized noble metal alloys, etc.), and the investigation of mechanisms involved in atomic and molecular cluster formation in polymeric media (nucleation, growth, aggregation, etc.) by optical and luminescence spectroscopy. He has authored 150 research articles published in international journals, ten patents, and many conference papers. He is the editor of two Wiley books devoted to metal-polymer nanocomposites and is a member of the editorial board of different scientific journals.

AL, PhD in ‘Materials and Structures Engineering,’ degree in chemical engineering, is currently a researcher at the Institute for Composite and Biomedical Materials - National Research Council (IMCB-CNR) of Naples. Her current scientific interests are related to the development of new methods to prepare nanostructured materials as polymer-embedded inorganic nanostructures. Furthermore, her interests include the design and development of advanced devices for electronic, optoelectronic, and energy storage application fields based on nanostructured materials. In particular, her work concerns the study of new chemical synthesis and the morphological-structural characterization of nanomaterials by electron microscopy (SEM, TEM) and also the X-ray powder diffraction (XRD) and optical spectroscopy techniques (UV-visible absorption and emission spectroscopy) to analyze the relation among chemical-physical properties and the nature, size, and shape of these nanomaterials. She has several dozen scientific papers on international journals and several participations at international conferences.

SDN received his BS degree in physics from the University of Naples “Federico II”, Italy, in 1982. From 1983 to 1987, he was a system analyst at Elettronica (Rome) and Alenia (Naples). Since 1988, he has been a staff researcher at the Institute of Cybernetics “E. Caianiello” of the National Research Council. Currently, he is a senior researcher at the SPIN Institute (Institute for Superconductors, oxides and other Innovative materials and devices), National Research Council (CNR). He has been a scientific coordinator of the research project ‘Imaging Techniques for Studying and Analyzing Microstructured Materials’ of the Department of Physics Sciences and Matter Technologies (DSFTM) of the National Research Council. He has been a coordinator of the research unit based at the Institute of Cybernetics in the framework of the Italian National Research FIRB program: Photonic Microdevices in Lithium Niobate. He has contributed to about 300 technical papers in peer-reviewed international journals, book chapters, and conference proceedings. He has served in program committees of several international conferences and has been a referee for various journals in the field of optics and theoretical physics. His research interests include the development of quantum methodologies to the description of coherent phenomena in many body systems, quantum tomography, theoretical modeling for studying dynamical effects in mesoscopic systems and nanostructured polymeric materials, electronic coherent transport in nonconventional superconductors and graphene, and interaction of optical and electron beams in nonlinear media and plasma.

CC is a senior researcher at the Instituto di Cibernetica “E. Caianiello” - CNR. His research centers on exploring the structural properties of superconductor thin films and their influence on the behavior and performances of Josephson devices based on both conventional and high Tc superconductors. This activity includes micro-Raman spectroscopy analysis and development of methods and processes for micro- and nanostructural engineering.

LN is the President of the National Research Council of Italy, a professor emeritus at the University of Naples “Federico II”, and an adjunct professor at the Universities of Connecticut in Storrs and Washington in Seattle. He has a prepost of the Schools of Science, Engineering, and Architecture of the University of Naples “Federico II”. He is the author of more than 500 papers in scientific journals and 35 patents and is also the editor of 15 books. He is a member of the editorial boards of many scientific journals. He was awarded the Society for the Advancement of Materials Technology (SAMPE) honor certificate, the ‘G. Dorsi’ and ‘Scanno’ prizes, and the gold medal of the Academy of the Forty. LN significantly contributed to the development of knowledge in the field of composite materials, rheology, energy and mass diffusion through polymers, and materials for biomedical application.

## Supplementary Material

Additional file 1 **Supporting information.** The file contains a schematic illustration of a carbon nanoscroll and the calculation of the arc length of a piece of spiral. Click here for file
