# Supplementary material for: A simple mechanical technique to obtain carbon nanoscrolls from graphite nanoplatelets
Source: Nanoscale Res Lett. 2013 Sep 30;8(1):403. doi: 10.1186/1556-276X-8-403 (PMC3849441; doi:10.1186/1556-276X-8-403)
Supplement: Additional file 1 — Supporting information. The file contains a schematic illustration of a carbon nanoscroll and the calculation of the arc length of a piece of spiral. [file 1556-276X-8-403-S1.doc]

**Supporting information**

The shape of a graphene sheet rolled up into a CNS with inner core radius $r_{0}$, outer radius *R* and interlayer spacing *t* can be approximately described by the Archimedean spiral $r\left(\theta\right)=r_{0}+\frac{t}{2\pi}\theta$ where $r$ and $\theta$ are the polar coordinates in the cross-sectional plane (fig. 1)

**Fig. 1 Schematic illustration of a carbon nanoscroll with inner core radius $r_{0}$ and outer radius *R*. The interlayer spacing is *t***

The arc length $L(R,r_{o},t)$ of the piece of spiral contained between the angles $\theta$=0 and $\theta$=$2\pi N$, where $N=\left(R-r_{0}\right)/t$ is the number of coils between the inner radius and the outer radius ( fig. 1), can be calculated explicitly in the following form

$L(R,r_{o},t)$=


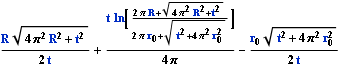
 (1)

According to our experimental data, the outer diameter is $D=2 R\sim100$nm Assuming an interlayer distance $t=0.34 nm$ and a length *L*=2.5 $\mu$m we get N=8.2 and inner radius $r_{0}$**=47.2 nm.** For a length 0.5 $\mu$m corresponding to the smallest observed nanoscroll we have N=1.6 and inner radius $r_{0}$**=49.45 nm**
